# Differences in Blood Eosinophil Level During Stable Disease and During Exacerbation of COPD and Exacerbation Risks

**DOI:** 10.1007/s00408-025-00792-9

**Published:** 2025-02-27

**Authors:** Wang Chun Kwok, Terence Chi Chun Tam, Chi Hung Chau, Fai Man Lam, James Chung Man Ho

**Affiliations:** 1 Department of Medicine, The University of Hong Kong, Queen Mary Hospital, 4/F, Professorial Block, 102 Pokfulam Road, Pokfulam, Hong Kong SAR China; 2 Tuberculosis and Chest Unit, Grantham Hospital, 125 Wong Chuk Hang Road, Aberdeen, Hong Kong SAR China

**Keywords:** COPD, Eosinophil, Eosinophil difference, Phenotype, COPD exacerbation

## Abstract

**Background:**

Although blood eosinophil count (BEC) has been extensively studied as a biomarker in chronic obstructive pulmonary disease (COPD), there remain challenges and controversy in using a single reading. It has not been determined whether the difference in BEC between baseline and that during an acute exacerbation of COPD (AECOPD) has any role in predicting subsequent AECOPD.

**Methods:**

A prospective study was conducted to investigate the possible role of differences in BEC from baseline to that during AECOPD to predict future AECOPD risk. The BEC difference was expressed as absolute eosinophil difference: BEC at index moderate-to-severe exacerbation (*E*_i_) – baseline BEC (*E*_0_).

**Results:**

Among 348 Chinese patients with COPD, 158 who experienced an index moderate-to-severe AECOPD were analyzed. Using the cut-off of 105 cells/µL for absolute eosinophil difference as determined by receiver operating characteristic (ROC) analysis, patients with absolute eosinophil difference ≥ 105 cells/µL had a shorter time to subsequent AECOPD with adjusted hazard ratio (aHR) of 1.68 (95% CI = 1.02–2.74; *p* = 0.040). They also had a higher annual number of subsequent AECOPD (2.49 ± 2.84/year vs 1.58 ± 2.44/year, *p* = 0.023). Similar findings were shown in the subgroup with stable-state baseline BEC < 300 cells/µL.

**Conclusion:**

Greater difference in BEC between baseline and upon moderate-to-severe AECOPD might be associated with shorter time to next AECOPD, as well as more episodes of subsequent AECOPD.

**Supplementary Information:**

The online version contains supplementary material available at 10.1007/s00408-025-00792-9.

## Background

Blood eosinophil count (BEC) has been extensively studied as a mean of phenotyping in chronic obstructive pulmonary disease (COPD) [1–6]. Eosinophilic airway inflammation has been shown to be present in stable COPD [7–11] and during acute exacerbations of COPD (AECOPD) [12–15]. Although higher BEC during stable disease was found to indicate a greater risk of AECOPD [16], the Scientific Committee of GOLD [17] suggested that BEC alone was not a useful biomarker of exacerbation risk in clinical practice [18]. They suggest that the potential usefulness of BEC as a predictor of future exacerbation risk is restricted to patients with a history of exacerbations. Exacerbation history remains the best predictor of future exacerbation risk [19]. Higher BEC at the time of AECOPD was also associated with adverse outcome [20]. Nonetheless this finding derived from a retrospective study that defined the adverse outcome as late readmission rate. On the contrary, other studies suggested that higher BEC upon AECOPD was associated with shorter hospital stay [21, 22]. Recently, a prediction score incorporating BEC to predict AECOPD risk has been developed [23]. Nonetheless there remain challenges and criticisms with regard to using a single BEC value in prognostication [24–26], especially when biological variation in BEC has been demonstrated in healthy individuals [27]. Desiree et al [26] reported that BEC varies significantly throughout the disease course of COPD. In that study, using a cut-off of 150 cells/μL to stratify high and low BEC values, 79% of patients would have discordant values. The discordance rate was 54% when 300 cells/μL was chosen as the cut-off [26]. A retrospective study suggested that the number of AECOPD was higher in patients with higher variability in BEC [28]. Another study suggested that patients with BEC > 150 cells/μL, especially predominantly increased BEC based on multiple measurements, had a lower risk of all-cause mortality [29]. A Korean study found that patients with persistently high BEC had better survival than those with persistently low BEC [30]. Our group also reported that variability in BEC during stable disease might predict AECOPD risk [31]. In the same study, applying the conventional cut-off for a single BEC measurement in COPD did not predict AECOPD risk.

In light of the controversies surrounding use of a single BEC, especially with the knowledge that BEC variability and differences might have better prognostic value, we conducted a prospective study of the difference in BEC at the time of moderate-to-severe exacerbation compared with that during a stable-state and its role in predicting future exacerbation risk.

## Methods

A prospective study was conducted at Queen Mary Hospital (QMH) and Grantham Hospital (GH). Both are regional hospitals and tertiary respiratory referral centers in Hong Kong. All Chinese patients aged at least 40 years, with at least 10 pack years smoking history, and COPD undergoing regular follow up at QMH or GH in the year 2021 were recruited. Patients with clinically stable COPD were recruited from the respiratory specialty clinic at QMH/GH during routine follow-up visits. A clinically stable-state was defined as being free from AECOPD and any systemic corticosteroid use for longer than 90 days. The diagnosis of COPD was confirmed by spirometry demonstrating post-bronchodilator airflow limitation with forced expiratory volume in one second/forced vital capacity [FEV_1_/FVC] ratio < 0.7, in line with the latest recommendation of the Global Strategy for Prevention, Diagnosis and Management of COPD Report [17]. Patients with co-existent asthma (compatible clinical history; previous physician diagnosis of asthma; with significant bronchodilator response on spirometry as a supporting criteria for cases in doubt), bronchiectasis (by radiological features on chest X-ray ± high-resolution computed tomography (HRCT)), interstitial lung disease (by chest X-ray ± HRCT) and other conditions that might alter BEC, such as hematological malignancies, hypereosinophilic syndrome and active parasitic infection, were excluded. Patients who died during the index AECOPD episode were also excluded. Written informed consent was obtained. History taking, physical examination and blood taking for complete blood count was performed at the time of recruitment. Demographic data, clinical data/investigations, and medication record were also recorded. Regular use of inhaled corticosteroid (ICS), long-acting beta-agonists (LABA), long-acting muscarinic antagonists (LAMA), theophylline and roflumilast was defined as continuous use for at least 12 months prior to recruitment.

The baseline BEC (E_0_) during a clinical stable-state in 2021 was recorded at the recruitment visit, at least 90 days from the last AECOPD and any systemic corticosteroid use. AECOPD was defined as an acute event characterized by worsening of respiratory symptoms beyond normal day-to-day variations with consequent change in medications. Symptoms included one or more of the following: [1] increased cough frequency and severity; [2] increased sputum volume and/or altered sputum character; [3] increased dyspnea that required medical attention and treatment [17]. Mild AECOPD was defined as AECOPD that was treated with short acting bronchodilators only. Moderate AECOPD was defined as AECOPD treated with short acting bronchodilators and oral corticosteroid ± oral antibiotics. Severe AECOPD was defined as AECOPD that required hospitalization or an emergency department visit [17]. The index AECOPD episode was defined as an AECOPD episode of moderate-to-severe severity. Patients continued to receive standard-of-care treatment from the primary team in charge after recruitment to the study. The patients were prospectively followed up in the respiratory/COPD specialty clinic at QMH/GH every 16–26 weeks until 30th October 2023 and monitored for symptoms, COPD control, presence of mild exacerbation, medication compliance, and the date of the first moderate-to-severe exacerbation. Investigators were alerted when patients experienced the index moderate-to-severe AECOPD episode at an ad hoc clinic visit or when they attended the emergency department or were hospitalized, and a blood sample obtained for measurement of BEC (*E*_i_) prior to administration of systemic corticosteroid. The BEC difference was expressed as the absolute difference between BEC during the moderate-to-severe exacerbation (*E*_i_) and baseline BEC (*E*_0_): *E*_i_−*E*_0_.

The primary outcome was the time to subsequent AECOPD after the index moderate-to-severe AECOPD episode. Secondary outcome was the number per year of subsequent AECOPD after the index episode.

This study was approved by The University of Hong Kong and Hospital Authority Hong Kong West Cluster Institutional Review Board (approval reference number: UW 21-172).

### Statistical Analysis

The demographic and clinical data are described as actual frequency, mean ± SD or median [inter-quartile range (25th–75th percentile)]. Baseline demographic and clinical data were compared between the two groups (with or without subsequent AECOPD after the index moderate-to-severe AECOPD episode) with independent t-tests or non-parametric tests where appropriate. To define the optimal cut-off value of absolute eosinophil difference to predict subsequent AECOPD, receiver operating characteristic (ROC) curve analysis was performed. Cox regression analysis was used to assess time to next AECOPD, based on this newly defined eosinophil difference cut-off. Kaplan–Meier analysis was used to estimate the cumulative event rates and stratified log-rank statistics to assess the effects of eosinophil difference during the follow-up period with respect to the composite endpoint of AECOPD. Negative binomial regression was used to estimate the association of eosinophil difference with number of subsequent AECOPD after the index episode. Age, gender, smoking status, FEV_1_, mMRC dyspnoea scale, Charlson comorbidity index, number of moderate-to-severe AECOPD in the year prior to subject recruitment, use of relevant medications (ICS, LAMA, LABA, theophylline and roflumilast), and vaccinations against influenza and *Streptococcus pneumoniae* were adjusted as potential confounders. Subgroup analyses were performed in patients with baseline BEC < 300 cells/µL and ≥ 300 cells/µL. Sensitivity analysis was performed on patients with or without late subsequent AECOPD, defined as those in whom AECOPD developed more than 15 days after the index episode. Statistical significance was determined at the level of *p* = 0.05 (2-sided test). All statistical analyses were done using the 28th version of SPSS statistical package.

## Results

A total of 348 Chinese patients with COPD were recruited, of whom 158 with an index moderate-to-severe AECOPD were analyzed. There were 18 patients who had a mild exacerbation only and who were excluded from analysis. The mean follow-up was 18.3 ± 9.2 months (range = 6.1–32.9 months).

### Baseline Characteristics

The mean age was 77.7 ± 9.3 years, with a male predominance (86.6%). The mean age at diagnosis of COPD was 70.2 ± 9.9 years. The mean FEV_1_ was 1.14 ± 0.51 L (53 ± 21% predicted). The median mMRC score was 2 [1–3]. The median BEC at stable-state and during the index moderate-to-severe AECOPD was 150 [70–350] and 280 [170–502] cells/µL, respectively. 109 patients had subsequent AECOPD after the index episode, with 9 having early subsequent AECOPD within 15 days and 100 having late subsequent AECOPD defined as occurrence > 15 days after the index episode [32].

Patients with a subsequent AECOPD were more symptomatic according to the mMRC dyspnoea scale, had worse lung function (FEV_1)_ and were more likely to be prescribed LABA and LAMA. The results are summarized in Table 1.

**Table 1:** Baseline demographic and clinical characteristics

	Whole cohort (*n* = 158)	No subsequent COPD exacerbation after index moderate-to-severe AECOPD (*n* = 49)	Had subsequent COPD exacerbation after index moderate-to-severe AECOPD (*n* = 109)	*p*-values
Age (years), mean ± SD	77.7 ± 9.3	76.9 ± 9.8	78.1 ± 9.1	0.51
Male	140 (86.6%)	45 (91.8%)	95 (87.2%)	0.39
Smoking status				0.07
Current smoker	31 (19.6%)	14 (28.6%)	17 (15.6%)	
Ex-smoker	127 (80.4%)	35 (71.4%)	92 (84.4%)	
mMRC dyspnoea scale, mean ± SD	1.72 ± 0.97	1.39 ± 0.73	1.97 ± 1.06	< 0.001*
FEV_1_ (L), mean ± SD	1.14 ± 0.51	1.35 ± 0.58	1.04 ± 0.44	0.002*
FEV_1_ (% predicted), mean ± SD	53 ± 21	59.8 ± 19.8	49.3 ± 20.9	0.003*
FVC (L), mean ± SD	2.33 ± 0.85	2.51 ± 0.92	2.25 ± 0.80	0.089
FVC (% predicted), mean ± SD	78.8 ± 23.2	84.8 ± 25.1	76.2 ± 22.0	0.070
FEV_1_/FVC ratio (%), mean ± SD	51.0 ± 17.9	57.7 ± 15.7	48.0 ± 18.0	< 0.001*
Bronchodilator reversibility (mL), mean ± SD	68 ± 79	77 ± 87	64 ± 74	0.44
Bronchodilator reversibility (%), mean ± SD	8.9 ± 19.3	10.1 ± 28.9	8.4 ± 12.6	0.73
Number of exacerbation(s) in the past 1 year, mean ± SD	0.71 ± 2.11	0.35 ± 1.21	0.87 ± 2.40	0.07
Blood eosinophil count at bassline (x cells/µL), median [25th–75th percentile]	150 [70–350]	210 [65–375]	140 [75–325]	0.43
Blood eosinophil count at index moderate-to-severe AECOPD x cells/µL), median [25th–75th percentile]	280 [170–502]	240 [140–500]	300 [195–505]	0.25
LABA use	144 (91.1%)	39 (79.6%)	105 (96.3%)	0.001*
LAMA use	149 (94.3%)	42 (85.7%)	107 (98.2%)	0.002*
ICS use	108 (68.4%)	29 (59.2%)	79 (72.5%)	0.10
Theophylline use	39 (24.7%)	11 (22.4%)	28 (25.7%)	0.66
Roflumilast use	16 (10.1%)	3 (6.1%)	13 (11.9%)	0.26
Received PCV	22 (13.9%)	4 (8.2%)	18 (16.5%)	0.16
Received PSV	67 (42.4%)	24 (49.0%)	43 (39.4%)	0.26
Received influenza vaccine	100 (63.3%)	28 (57.1%)	72 (66.1%)	0.28

*AECOPD* Acute exacerbation of chronic obstructive pulmonary disease, *SD* standard deviation, *mL* milliliter, * statistically significant, *FEV*_*1*_ forced expiratory volume in one second, *FVC* forced vital capacity, *CAT* COPD Assessment Test, *mMRC* Modified Medical Research Council, *LABA* long-acting beta-agonists, *LAMA* long-acting muscarinic antagonists, *ICS* inhaled corticosteroid, *PCV* pneumococcal conjugated vaccine, *PSV* pneumococcal polysaccharide vaccine

### Determination of Eosinophil Difference Cut-Off Value

**Fig. 1:**
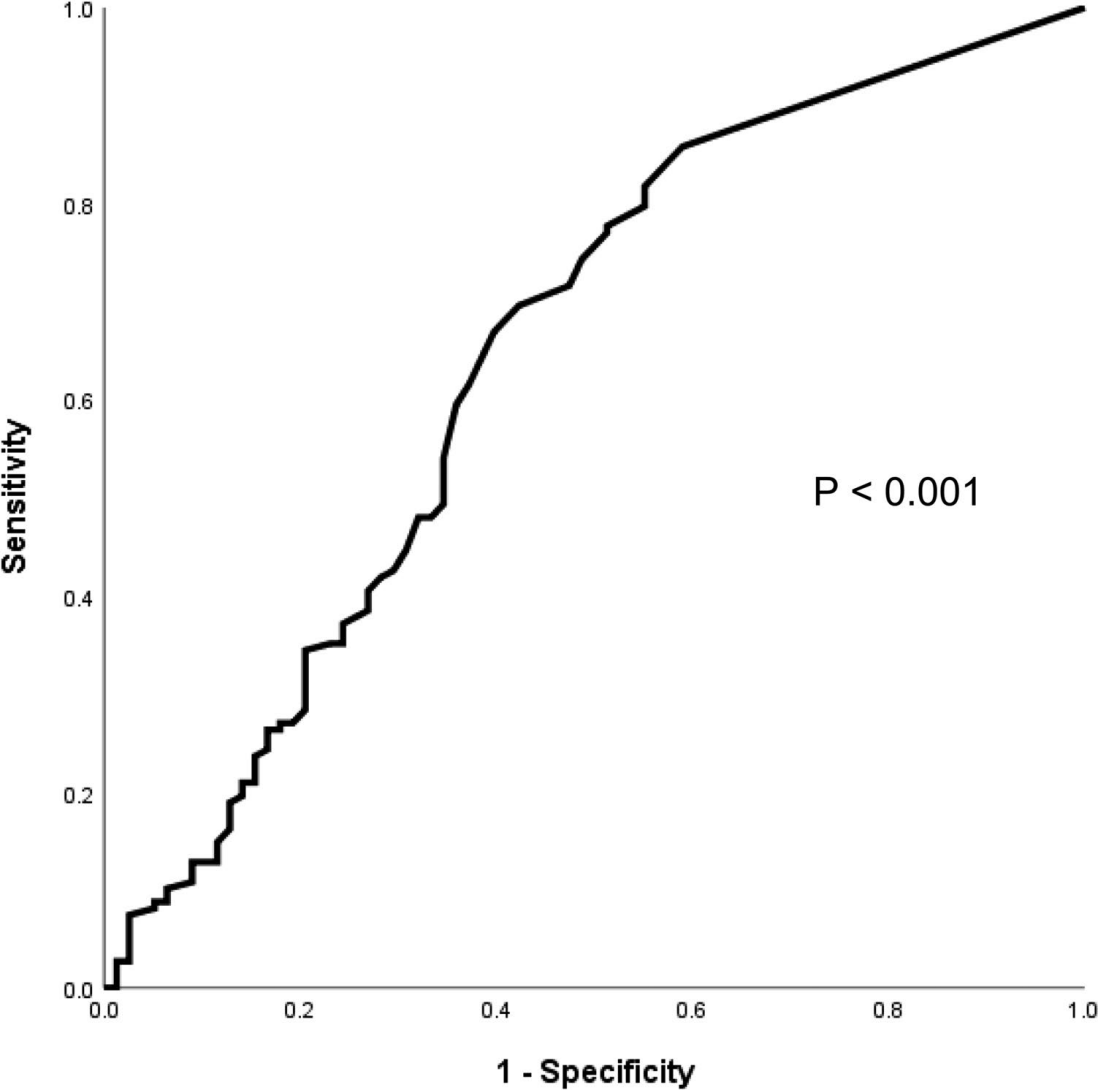
Receiver Operating Curve (ROC) for absolute eosinophil difference and risk of subsequent AECOPD

Results of ROC analysis revealed that a cut-off at 105 cells/µL (absolute eosinophil difference) had sensitivity of 64.3% and specificity of 61.3% in predicting subsequent AECOPD, with area under the curve (AUC) of 0.65 (95% confidence interval [CI] = 0.57–0.73; *p* < 0.001) (Fig. 1).

The cut-off for high sensitivity and high specificity for absolute eosinophil difference was 85 cells/µL (sensitivity 72.5%, specificity 54.0%) and 255 cells/µL (sensitivity 36.7%, specificity 82.0%) respectively.

### Time to Subsequent AECOPD Stratified by Absolute Eosinophil Difference

**Fig. 2:**
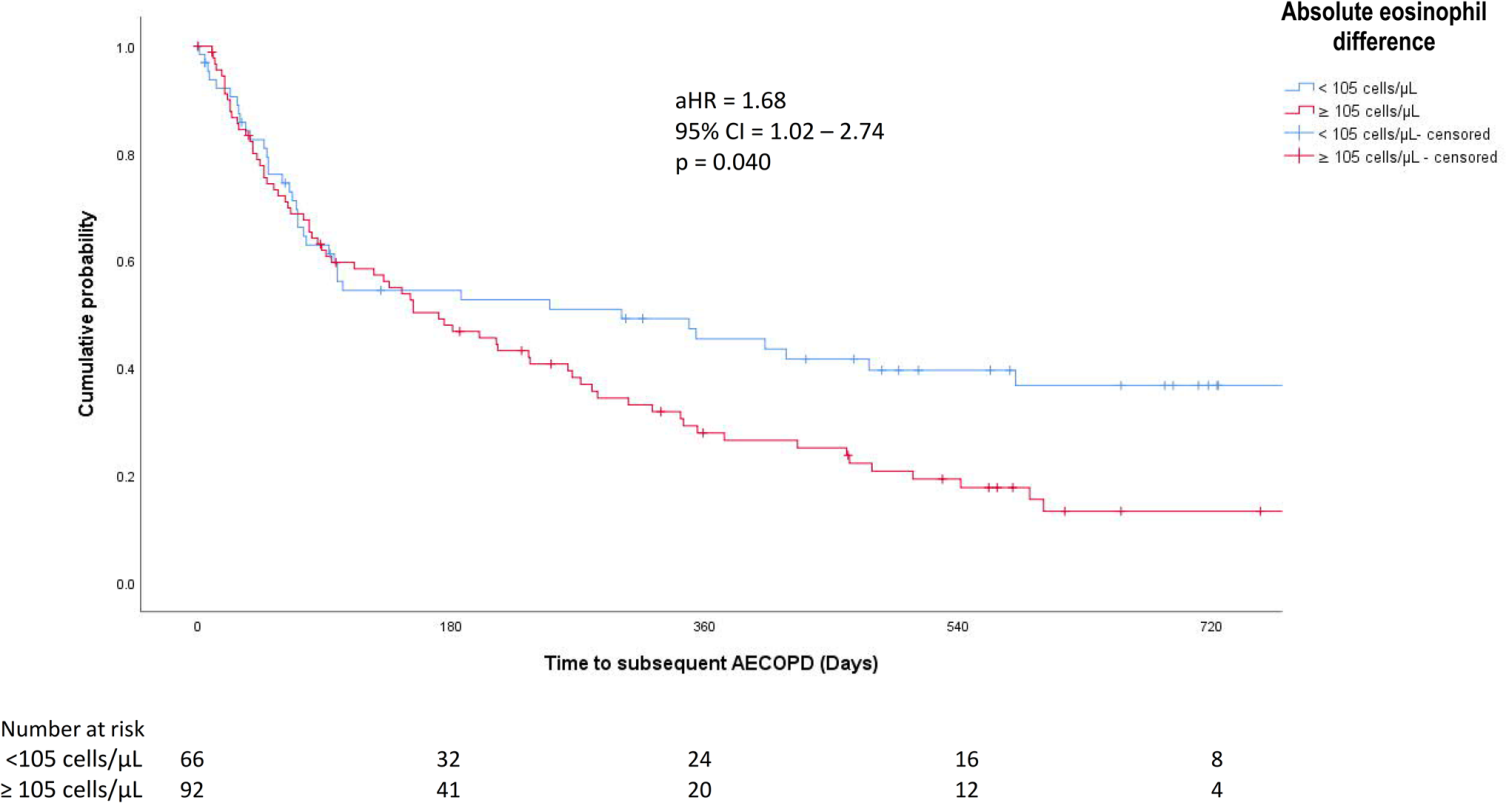
Time to subsequent AECOPD among patients with absolute eosinophil difference < or ≥ 105 cells/µL

Patients with absolute eosinophil difference ≥ 105 cells/µL had a shorter time to subsequent AECOPD with a median of 171 days (95% CI = 100–242 days) compared with 301 days (95% CI = 0–605 days) for patients with absolute eosinophil difference < 105 cells/µL. The hazard ratio (HR) was 1.53 (95% CI = 1.03–2.28; *p* = 0.036). The adjusted HR (aHR) was 1.68 (95% CI = 1.02–2.74; *p* = 0.040), suggesting patients with absolute eosinophil difference ≥ 105 cells/µL had increased risks for subsequent AECOPD (Fig. 2).

### Annual Number of Subsequent AECOPD After the Index Moderate-to-Severe AECOPD

The annual number of subsequent AECOPD after the index moderate-to-severe episode in patients with absolute eosinophil difference < 105 cells/µL versus ≥ 105 cells/µL was 1.58 ± 2.44 versus 2.49 ± 2.84 respectively (*p* = 0.046 by univariate analysis; *p* = 0.023 by multivariate analysis).

### Subgroup Analysis in Patients with Baseline BEC < 300 Cells/µL

There were 111 patients with baseline BEC < 300 cells/µL. Results of ROC analysis revealed that a cut-off at 105 cells/µL (absolute eosinophil difference) had a sensitivity of 69.1% and specificity of 56.6% in predicting subsequent AECOPD, with AUC of 0.61 (95% CI = 0.51–0.71; *p* = 0.02) (Supplementary Figure S1).

The median time to subsequent AECOPD was shorter in those with absolute eosinophil difference ≥ 105 cells/µL versus < 105 cells/µL [153 days (95% CI = 48–258) versus 187 days (95% CI = 0–442)]. The aHR was 1.86 (95% CI = 1.06–3.23), *p* = 0.030, suggesting that patients with absolute eosinophil difference ≥ 105 cells/µL had increased risks of subsequent AECOPD in this subgroup (Supplementary Figure S2).

The absolute eosinophil difference did not predict annual number of subsequent AECOPD after the index moderate-to-severe AECOPD (*p* > 0.05) in this subgroup. The mean annual number of subsequent AECOPD was 2.56 ± 2.86 and 2.03 ± 2.78 among patients with absolute eosinophil difference ≥ 105 cells/µL and < 105 cells/µL, respectively, *p* = 0.34.

### Subgroup Analysis in Patients with Baseline BEC ≥ 300 Cells/µL

There were 47 patients with baseline BEC ≥ 300 cells/µL. ROC analysis could not identify an optimal cut-off for absolute eosinophil difference. In this subgroup, absolute eosinophil difference did not predict the time to subsequent AECOPD or annual number of subsequent AECOPD after the index episode (*p* > 0.05). The mean annual number of subsequent AECOPD was 1.83 ± 2.70 and 0.96 ± 1.77 among patients with absolute eosinophil difference ≥ 105 cells/µL and < 105 cells/µL, respectively, *p* = 0.23.

### Sensitivity Analysis on Late Readmissions

Sensitivity analysis was performed on patients with or without late subsequent AECOPD, defined as AECOPD that developed more than 15 days after the index episode. Those with early subsequent AECOPD were excluded.

The aHR was 2.06 (95% CI = 1.08–3.92), *p* = 0.028 for absolute eosinophil difference ≥ 105 cells/µL, suggesting that patients with absolute eosinophil difference ≥ 105 cells/µL had increased risks of subsequent late AECOPD.

## Discussion

In this study, a high difference in BEC between baseline and that during a moderate-to-severe AECOPD, as measured by absolute eosinophil difference, was shown to be associated with shorter time to next AECOPD and more subsequent AECOPD. The results were also more obvious among the subgroup with baseline BEC < 300 cells/µL. The findings concur with previous reports that differences in BEC may play an important role in prognostication [31].

According to past studies, the use of a single value BEC, during stable-state or upon exacerbation, appears inadequate to predict the outcome of COPD since it may not comprehensively reflect the patient’s inflammatory profile. It is well-known that the BEC varies within subjects with even greater inter-subject variation among healthy individuals [27]. As such, variability in baseline BEC has been shown to be superior to a single value BEC to predict future risk of AECOPD [31]. We hypothesized that the differences in BEC from baseline to moderate-to-severe AECOPD could be even better in predicting time to next AECOPD and AECOPD frequency.

The findings from this study were consistent with our previous work [31] that established that among the conventional non-eosinophilic subgroup, we may be able to further stratify patients into different subgroups with different endotypes and risks of AECOPD. A Korean study proposed definitions of variability as persistently < 300 cell/μL (persistently low), variable above and below 300 cell/μL (variable), and persistently ≥ 300 cell/μL (persistently high) [30]. As in this study and prior studies, among patients with an eosinophilic phenotype defined using a single BEC cut-off, the differences in BEC either at stable-state or between stable-state and moderate-to-severe AECOPD may not have any major impact on subsequent AECOPD risks since these patients already have a baseline heightened AECOPD risk. Nonetheless among the conventional non-eosinophilic subgroup, low-low and low–high subgroups may behave differently. The low-low subgroup of patients who lack eosinophil change may behave similarly to the conventional neutrophilic COPD patients while the low–high subgroup with high degree of eosinophil change may be phenotypically closer to the conventional eosinophilic group. This corroborates the observed phenomenon of differences and discordance in eosinophil count both during a stable-state as well as between stable-state and exacerbation and the association with future AECOPD risks.

Although the variability at baseline represents a stable-state BEC variation, the differences between stable-state and exacerbation may indicate both the inflammatory pattern at the time of exacerbation as well as the severity of inflammation. We hypothesize that higher eosinophil differences between stable-state and exacerbation may provide indirect evidence that the underlying pathophysiology of the AECOPD is driven by eosinophilic inflammation that may eventually lead to an increased risk of subsequent AECOPD. We also observed a non-significant increase in length of stay in the index AECOPD in this cohort that may also represent the same phenomenon.

The potential mechanisms to account for the differences in blood eosinophil count between baseline and AECOPD may be related to the underlying trigger or mechanism of AECOPD. MacDonald et al. reported that BEC was significantly higher in the AECOPD group not associated with infection [33]. Another study also revealed that patients with eosinophilic AECOPD were less likely to have evidence of bacterial infection [34]. Other factors that were postulated to affect stability of BEC include smoking status [35] and air pollution [36]. The exact aetiologies for a high and low absolute eosinophil difference could be a combination of these factors.

The findings from this study, together with other reports about eosinophil variability and differences, support continuous research and phenotyping among patients with COPD. Although a single value BEC is easy to use, it lacks precision. Incorporation of eosinophil variability and differences, both at baseline and between baseline and exacerbation, may help to further stratify patients into various risk groups, particularly those who are currently classified as a non-eosinophilic phenotype.

Our study has several limitations. First, the study was conducted at two tertiary centeres. It may have missed patients with milder COPD managed in the primary care setting. Nonetheless these patients are less likely to experience AECOPD, the primary outcome of our study. Second, of the 348 patients with COPD recruited, 158 had AECOPD and were included in the analysis, a relatively small sample that may have affected the study’s overall validity and generalizability. This was reflected by the AUC of ROC being 0.65 only and the 95% CI in HR close to 1. A small sample size would have limited the statistical power of our study. A larger scale study should be conducted to validate the findings. The majority of patients recruited were male, related to the relatively low smoking prevalence among females in Hong Kong and relatively low prevalence of female COPD. In this study, the *E*_i_ was measured at the index AECOPD but not subsequent AECOPD. Ideally, measuring multiple *E*_0_ and *E*_i_ would help determine whether this observed phenomenon is genuine or related to random variations over time. This would require a longer study duration. Also, multiple E_0_ may not be available for patients with repeat early subsequent AECOPD since exacerbations may recur before they achieve a between exacerbation clinically stable-state. To overcome this limitation, a larger sample size will be needed. Another limitation is on the follow-up duration. While the mean follow-up duration is 18.3 months, there were patients who had follow-up less than 1 year. The short follow-up duration for some of the included patients may subject them to have AECOPD affected by seasonal factors such as viral infection, weather change and air pollution.

## Conclusion

Higher differences in BEC between baseline and upon moderate-to-severe AECOPD might be associated with shorter time to next AECOPD, as well as more episodes of subsequent AECOPD.

## Supplementary Information

Below is the link to the electronic supplementary material.

Supplementary file1 (TIF 489 KB) Supplementary Figure S1 Receiver Operating Curve (ROC) for absolute eosinophil difference and risk of subsequent AECOPD among subgroup with baseline BEC < 300 cells/µL

Supplementary file2 (TIF 3664 KB) Supplementary Figure S2 Time to subsequent AECOPD among patients with absolute eosinophil difference < or ≥ 105 cells/µL among subgroup with baseline BEC < 300 cells/µL

## Data Availability

Dataset supporting the conclusion of this article is included within this article and no additional data will be provided. Research data is not shared.
