# Supplementary figures and images for: Differences in Blood Eosinophil Level During Stable Disease and During Exacerbation of COPD and Exacerbation Risks
Source: Lung. 2025 Feb 27;203(1):37. doi: 10.1007/s00408-025-00792-9 (PMC11868221; doi:10.1007/s00408-025-00792-9)

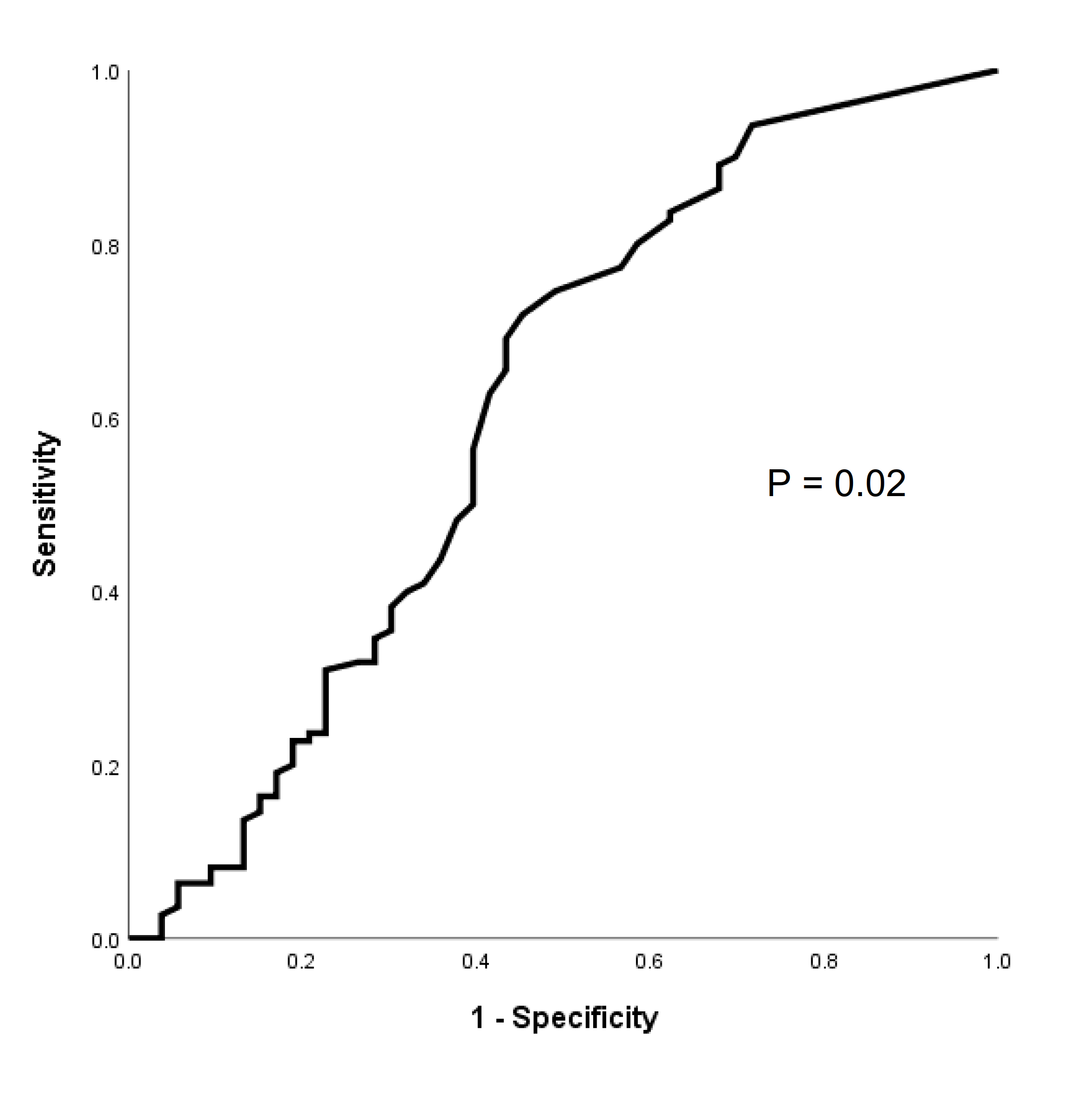

Supplement: Supplementary file 1 — Supplementary file1 (TIF 489 KB) Supplementary Figure S1 Receiver Operating Curve (ROC) for absolute eosinophil difference and risk of subsequent AECOPD among subgroup with baseline BEC < 300 cells/µL [file 408_2025_792_MOESM1_ESM.tif]

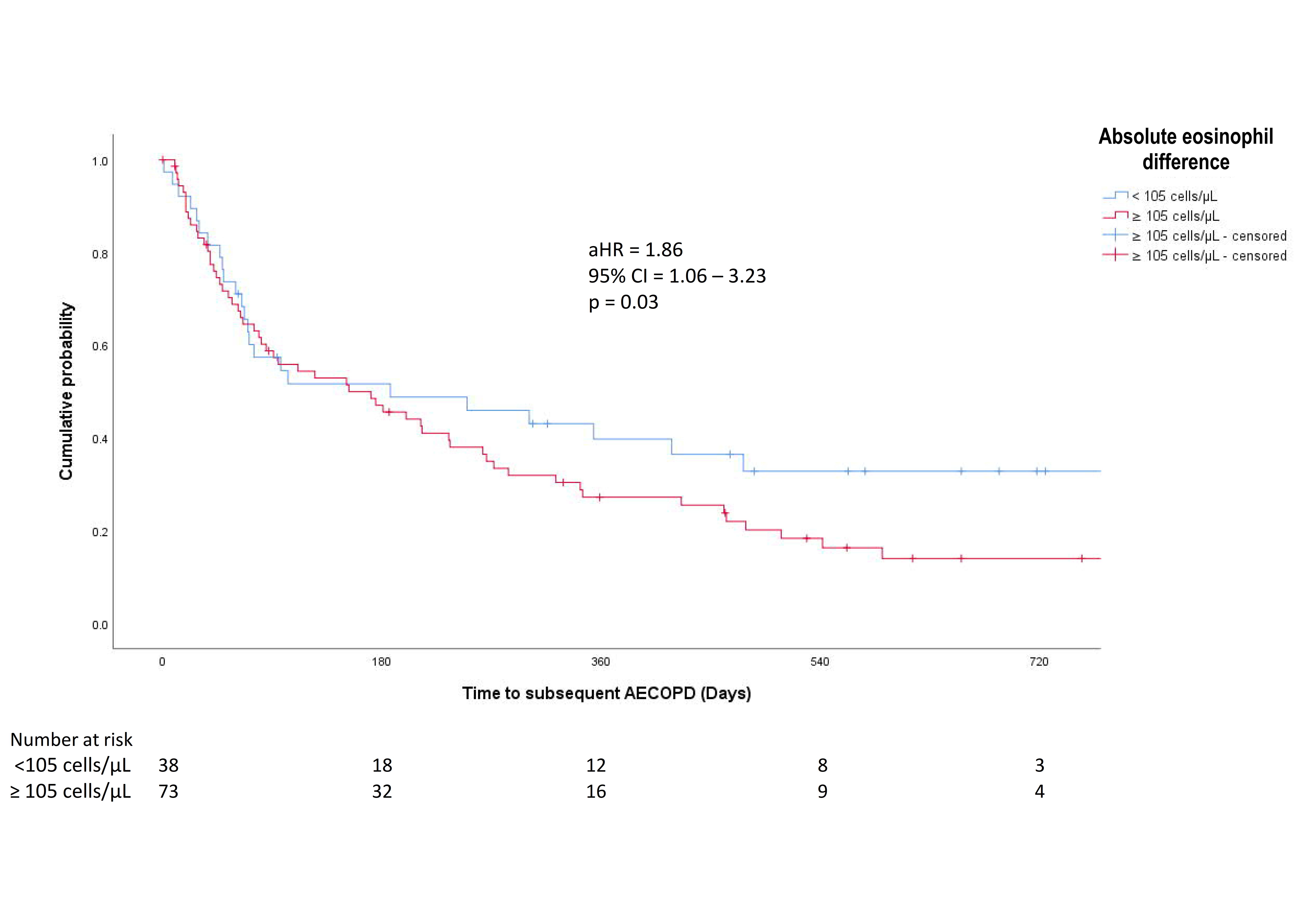

Supplement: Supplementary file 2 — Supplementary file2 (TIF 3664 KB) Supplementary Figure S2 Time to subsequent AECOPD among patients with absolute eosinophil difference < or ≥ 105 cells/µL among subgroup with baseline BEC < 300 cells/µL [file 408_2025_792_MOESM2_ESM.tif]
